
JOURNAL INFORMATION
==============================
NLM Title Abbreviation: ScientificWorldJournal
Journal ID: ScientificWorldJournal
NLM Journal ID: 101131163
Journal ID: TSWJ

The Scientific World Journal

ISSN: 2356-6140
EISSN: 1537-744X
Publisher: Wiley

ARTICLE INFORMATION
==============================
PMCID: PMC6009233
PMID: 29973819
DOI: 10.1100/tsw.2002.45
Article ID: 248978
Article version: 1
Subjects: Short Report

Protein Interactions Within the Cytoplasmic Dynein Complex as Probed by Mass Spectrometry and Nmr

Barbar Elisar *
Makokha Moses
Hare Michael
Department of Chemistry and Biochemistry, Ohio University, Athens, Ohio 45701, USA
*Elisar Barbar: barbar@ohio.edu
Publication date: 2002
Electronic publication date: 2002 Mar 12
Volume: 2
First page: 95
Last page: 96
Copyright: Copyright © 2002 Elisar Barbar et al.
Copyright year: 2002
License: This is an open access article distributed under the Creative Commons Attribution License, which permits unrestricted use, distribution, and reproduction in any medium, provided the original work is properly cited.
License URL: https://creativecommons.org/licenses/by/3.0/

Funding: National Institutes of Health GM60969 American Cancer Society

==============================
Advances in Gene Technology: The Genome and Beyond –
Structural Biology for Medicine (Proceedings of the 2002 Miami
*Nature Biotechnology* Winter Symposium)
*TheScientificWorld* 2002, 2(S2), 95–96
ISSN 1532-2246; DOI 10.1100/tsw.2002.45

### PROTEIN INTERACTIONS WITHIN THE CYTOPLASMIC DYNEIN COMPLEX AS PROBED BY MASS SPECTROMETRY AND NMR

Elisar Barbar*, Moses Makokha, and Michael Hare
Department of Chemistry and Biochemistry, Ohio University, Athens, Ohio 45701
*barbar@ohio.edu

**INTRODUCTION.** Cytoplasmic dynein is a principle motor for minus-end directed transport along microtubules[1]. It functions to position organelles and other materials within the cell, and plays an essential role in the separation of chromosomes. Cytoplasmic dynein is a large (1.2 MDa) complex made up of two heavy chain subunits linked by stalk-like domains to a common base. The heavy chains contain the ATP hydrolysis and microtubule binding sites required for motility. Located at the base are a number of subunits thought to regulate the assembly of the complex and its attachment to appropriate cargo. The intermediate chain IC74 forms a key intermediary in the complex, as it is known to associate with the heavy chain and the essential accessory complex dynactin as well as other subunits in the base. We have found that two light chains, LC8 and Tctex-1, attach to the N-terminal domain of IC74 (N-IC74). Here, the interactions between IC74 and the light chains have been characterized by limited proteolysis and mass spectrometry and by NMR spectroscopy.

**METHODS.** The interactions of LC8, Tctex-1 and the N-IC74 from *Drosophila melanogaster* were characterized *in vitro* by affinity methods, limited proteolysis, and circular dichroism spectroscopy. Limited digestion of N-IC74 in the presence and absence of LC8, Tctex-1 or both localized binding of the light and intermediate chains to specific segments in the protein. These interactions were further investigated by heteronuclear NMR.

**RESULTS.** Limited tryptic digestion and mass spectrometry[2] localized binding of Tctex-1 to the vicinity of K104 and K105, and localized binding of LC8 to the region downstream of K130. Circular dichroism, fluorescence, sedimentation velocity and proteolysis studies indicate that N-IC74 has limited secondary and tertiary structure at near physiological solution conditions. Upon addition of LC8, N-IC74 becomes more ordered. This conformational change is reflected in increased global protection of N-IC74 from proteolytic digestion following the interaction, and in a significant change in the CD signal consistent with a shift from random coil to a more helical conformation. The sequence of IC74 that is responsible for the increase in structure upon binding was identified by heteronuclear NMR using IC74 constructs that contain the binding site, and regions of the protein predicted to fold into a coiled coil structure.

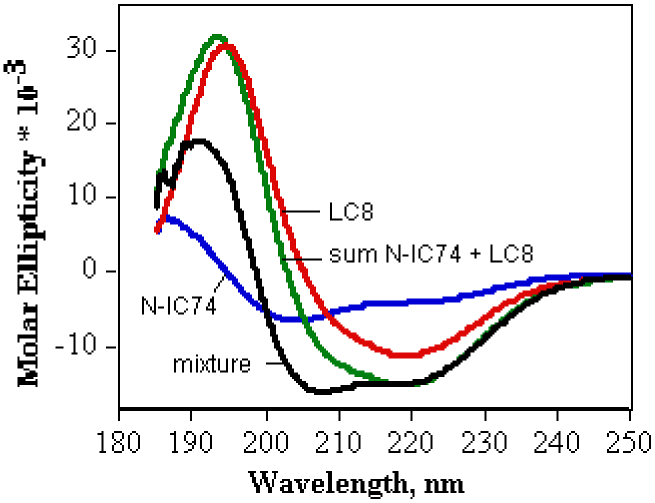

**FIGURE 1.** CD spectra of N-IC74 (blue), LC8 (red), and a mixture of the two (equimolar concentrations). The spectrum in green is computed from the sum of individual spectra of the two (model for no interaction). The spectrum of the mixture indicates an increase in helical conformations.

It is interesting to note that while the sequence of N-IC74 is not highly conserved among species (Drosophila N-IC74 has 23% homology with Dictyostelium, 30% with mouse), the disorder predicted from their sequences *is* conserved. We speculate that the disorder of this domain is a determinant of its specific interactions, and that a key event in formation of an active dynein complex is the simultaneous ordering of the IC74 structure and its binding with LC8.

**ACKNOWLEDGMENT.** This work was supported by National Institutes of Health Grant GM60969 (to E.B.), and American Cancer Society (to M.H.)

**REFERENCES**

1. King, S.M. (2000) Biochim. Biophys. Acta Mol. Cell Res. 1496, 60–75.
2. Spolaore, B., Bermejo, R., Zambonin, M., and Fontana, A. (2001) Biochemistry 40, 9460–9468.
